# Mechanisms That Activate 26S Proteasomes and Enhance Protein Degradation

**DOI:** 10.3390/biom11060779

**Published:** 2021-05-22

**Authors:** Alfred L. Goldberg, Hyoung Tae Kim, Donghoon Lee, Galen Andrew Collins

**Affiliations:** 1 Department of Cell Biology, Harvard Medical School, 240 Longwood Avenue, Boston, MA 02115, USA; donghoon_lee@hms.harvard.edu (D.L.); galen_collins@hms.harvard.edu (G.A.C.); 2 Neuroscience Research Center, GENUV Inc., Seoul 04520, Korea; karl@genuv.com

**Keywords:** ubiquitin–proteasome system, Usp14, Rad23, UBL-domain-containing proteins, ZFAND5, PKA, PKG

## Abstract

Although ubiquitination is widely assumed to be the only regulated step in the ubiquitin–proteasome pathway, recent studies have demonstrated several important mechanisms that regulate the activities of the 26S proteasome. Most proteasomes in cells are inactive but, upon binding a ubiquitinated substrate, become activated by a two-step mechanism requiring an association of the ubiquitin chain with Usp14 and then a loosely folded protein domain with the ATPases. The initial activation step is signaled by Usp14’s UBL domain, and many UBL-domain-containing proteins (e.g., Rad23, Parkin) also activate the proteasome. ZFAND5 is a distinct type of activator that binds ubiquitin conjugates and the proteasome and stimulates proteolysis during muscle atrophy. The proteasome’s activities are also regulated through subunit phosphorylation. Agents that raise cAMP and activate PKA stimulate within minutes Rpn6 phosphorylation and enhance the selective degradation of short-lived proteins. Likewise, hormones, fasting, and exercise, which raise cAMP, activate proteasomes and proteolysis in target tissues. Agents that raise cGMP and activate PKG also stimulate 26S activities but modify different subunit(s) and stimulate also the degradation of long-lived cell proteins. Both kinases enhance the selective degradation of aggregation-prone proteins that cause neurodegenerative diseases. These new mechanisms regulating proteolysis thus have clear physiological importance and therapeutic potential.

## 1. Overview of Proteasome Activity

For many years, our lab has focused on furthering our understanding of the 26S proteasome, both because of its key role in intracellular proteolysis and because of its intriguing molecular mechanisms. The efficient degradation of cell proteins by the ubiquitin–proteasome system (UPS) depends on its capacity to selectively bind ubiquitinated proteins, to disassemble ubiquitin chains, to unfold a large variety of proteins and translocate them into the 20S core proteasome, and to release small peptide products. All of these steps have to be precisely timed and integrated to ensure the rapid clearance of misfolded and regulatory proteins, as well as the slower hydrolysis of more stable cell constituents. Despite the dramatic recent advances in our knowledge of these processes obtained by cyro-electron microscopy of the 26S complex and through biochemical and biophysical studies of its multistep mechanism, many key features of the 26S proteasome’s multistep mechanism are not yet fully understood. Further knowledge about these integrated mechanisms is not only of basic biochemical interest, but also should illuminate our understanding of proteotoxic disease in light of the growing evidence in many neurodegenerative disease models that proteasome function is impaired due to the accumulation of aggregation-prone proteins. The resulting defects in protein degradation most likely accelerate the further accumulation of misfolded proteins, which must interfere with normal cell function.

This article reviews a number of important new insights about how the 26S proteasome’s activities are regulated that have emerged from our lab’s recent studies. A key technical development enabling many of these findings was our developing a one-step affinity purification method for gentle isolation of 26S proteasomes (the UBL-UIM method) [1]. This approach did not require genetic manipulation of the cells or tissues, and the resulting 26S preparations exhibited many regulatory properties that are lost in most multistep chromatographic methods. Although these studies were initiated to clarify the 26S proteasome’s multistep mechanism, these findings have altered our views of proteasome function and heterogeneity and have led to surprising new insights into how protein degradation is regulated in vivo. The initial, key observation made by Andreas Peth was that the 26S proteasome’s peptidase actively increases when it binds a ubiquitinated protein [2]. In other words, the proteasome functions as a “protease on-demand”, which becomes active only upon interaction with an appropriate substrate. Thus, in the cell, in the absence of a substrate, its capacity for proteolysis and rate of ATP hydrolysis are low, which must be of advantage to the organism in preventing unnecessary ATP consumption and potentially harmful unregulated proteolysis.

Surprisingly, this initial activation of the proteasome was not triggered by the interaction of the ubiquitinated substrate with one of the known receptors for ubiquitin chains (Rpn10, Rpn1, Rpn13), but instead by its binding to the proteasome-associated DUb, Usp14 [2,3], which, as we showed, plays a critical role in regulating proteasomal function [4,5]. Prior analysis of the yeast homolog of Usp14, Ubp6, had shown that Ubp6 functions normally as an allosteric inhibitor of the proteasome [6]. However, our data indicated that upon binding a ubiquitin chain, Usp14/Ubp6 switches its role and becomes an activator of the 26S proteasome, leading to its enhanced capacity to digest small peptides [2]. In addition, if an unfolded polypeptide chain is present on the proteasome, as is typical when a ubiquitinated substrate binds, ATP hydrolysis also rises and drives proteasome function in degrading the ubiquitin conjugates [7]. The increased ATP hydrolysis leads to tighter (probably irreversible) binding of the polypeptide, as is essential for processive degradation. This two-step activation mechanism helps to explain the selectivity of the proteasome and tightly links its three main enzymatic activities, deubiquitination, ATPase, and proteolysis.

The further dissection of Usp14’s activation mechanism by Hyoung Tae Kim led to the important finding that this activation upon substrate binding was mediated by Usp14’s UBL domain, whose movement upon substrate binding to Usp14’s active site seemed to cause activation [5]. In fact, this domain by itself can activate the 26S and mimic substrate binding. Galen Collins then went on to show that many, perhaps even most, of the cell proteins bearing a UBL domain can also activate the proteasome [8]. Many such UBL-domain-bearing proteins were known to bind to the proteasome. Our new finding was that this large family of UBL-containing proteins also activates the 26S and thus appears to enhance the likelihood of proteolysis. For example, when a shuttling factor such as Rad23 delivers a ubiquitinated substrate to the 26S proteasome, it also causes proteasome activation and can thus facilitate substrate degradation. These studies thus indicate two modes of proteasome activation (i.e., by the direct binding of ubiquitinated substrate or via binding a shuttling factor) that appear to function during each round of proteolysis in vivo and are probably maintained while the ubiquitinated protein is being degraded [8]. Our related studies had indicated that if a small ubiquitinated protein is bound directly to the proteasome, its hydrolysis requires around 20–30 s, but with tightly folded or larger polypeptides, the time required for degradation was longer [9]. The duration of the degradative process with a shuttling factor present and the duration of the activated state are unclear.

We have also studied in depth and describe below a very different type of proteasome activator, ZFAND5, a Zn finger protein, whose effects on proteasome activity raise intriguing mechanistic questions and are of clear biological importance [10]. ZFAND5 functions in atrophying muscles, where it enhances the ability of the 26S proteasome to hydrolyze ATP, peptides, and ubiquitinated proteins [11]. As a result, it also increases the muscle cell’s capacity for proteolysis and thus is critical in the atrophy process. ZFAND5 appears unique in multiple ways, but there may exist many other unidentified proteins that enhance in a similar manner proteasome function under special physiological conditions.

Finally, we have described a very different, but physiologically very important, type of 26S proteasome activation, through phosphorylation of a proteasome subunit. A number of protein kinases have been reported to modify the 26S proteasome and even to alter its activity [12,13], but for only three have effects on rates of intracellular protein degradation been clearly demonstrated: DYRK2, Protein Kinase A (PKA), and Protein Kinase G (PKG). It is noteworthy that these three protein kinases are active under distinct physiological conditions: DYRK2 during the S-M phase of the cell cycle, PKA in response to treatments that raise levels of cAMP, and PKG in response to treatments that raise cGMP levels [14,15,16]. Moreover, they appear to modify distinct proteasome components and to affect the degradation of different cell proteins, as discussed below. Our findings that PKA and PKG, which regulate a large diversity of physiological processes, also activate the proteasome and accelerate the degradation of many cell proteins within minutes represent new aspects of their function [16,17]. These findings have raised many fundamental questions and even opened up new opportunities for therapeutic interventions.

It is of obvious interest to understand how the phosphorylation of different proteasome components by DYRK2, PKA, or PKG can stimulate the proteasome’s enzymatic activities and to learn how the resulting proteasome activation influences the degradation of different cell proteins. Proteasome activation by cAMP or cGMP leads to an enhanced capacity of the cell to degrade many short-lived cell proteins, including the clearance of various aggregation-prone proteins that are associated with human disease [15,16,18]. Thus, pharmacological treatments that raise cAMP or cGMP, enhancing the degradation of the misfolded, mutated proteins, represent very promising new approaches to combat proteotoxic diseases, as discussed below. Finally, these findings have altered the way we should view the ubiquitin–proteasome system’s functioning in mammalian tissues. It has long been assumed that the cell’s capacity to eliminate misfolded potentially toxic proteins is fixed or declines with age [19]. Instead, these studies demonstrated that the cell’s degradative capacity can be rapidly and transiently activated in vivo by these (and probably other) signal transduction systems. The physiological significance of such regulation and its potential medical applications are still largely unexplored.

## 2. Proteasome Activation by Ubiquitin Conjugates through Usp14

**Figure 1:**
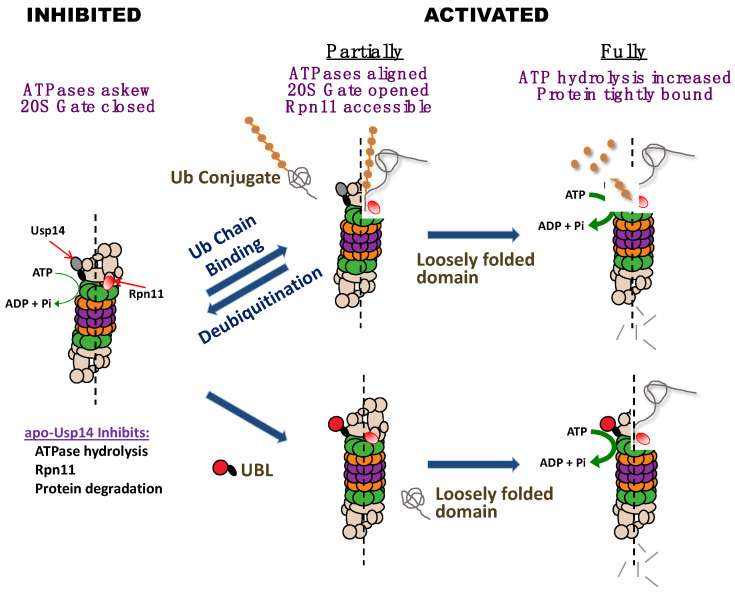
26S proteasomes inhibited by Usp14 become activated either upon ubiquitin conjugate binding to Usp14 or upon binding another cell protein containing a UBL domain. In the absence of a ubiquitin conjugate, Usp14 inhibits several proteasome activities: peptide hydrolysis (due to the misalignment of ATPases and the closed 20S gate), ATP hydrolysis, Rpn11-mediated de-ubiquitination, and consequently protein degradation. However, upon binding to a ubiquitin conjugate, Usp14, through its UBL domain, activates proteasomes: the ATPases align, the 20S gate opens, and Rpn11 becomes accessible to substrates. If a loosely folded domain is also present in the substrate, proteasomes become fully active, ATP hydrolysis increases, and the protein substrate is bound more tightly, leading to processive degradation. Alternatively, the binding to the proteasome of a protein containing a UBL domain (e.g., a UBL-UBA shuttling factor) stimulates peptide hydrolysis. Full activation occurs if, in addition, there is present a protein with a loosely folded domain.

During our efforts to understand how substrate de-ubiquitination on the proteasome may be linked to the ATP-dependent proteolytic steps, Andreas Peth found that the binding of a ubiquitin conjugate to the proteasomal deubiquitinating enzymes, Usp14/Ubp6 or Uch37/UchL5, allosterically activates the proteasome’s degradative mechanism [2,3]. This stimulatory role of Usp14 requires occupancy of its active site by either a ubiquitin chain or by the transition state inhibitor, ubiquitin aldehyde. By contrast, free ubiquitin has no stimulatory effect. This interaction of a substrate with Usp14’s active site, and not its initial binding to the 19S ubiquitin receptors, enhances the entry of substrates into the 20S and also increases ATP hydrolysis if the protein substrate contains a loosely folded domain (Figure 1) [3].

These two structural requirements for ATPase activation, a ubiquitin chain and a loose polypeptide domain, do not have to be covalently linked as they are in a ubiquitinated protein, because, on the proteasome, they interact with different parts of the 19S complex, the ubiquitin chain with Usp14 and the polypeptide with the ATPase ring. This two-step activation mechanism also accounts for the important findings by Matouschek and colleagues that proteasomal degradation of a ubiquitinated substrate requires both substrate ubiquitination and the presence of an unfolded domain in it [20,21].

The finding that Usp14/Ubp6 is a central regulator of 26S function (see Figure 1) has been further supported by the recent cryo-electron microscopic studies by the labs of Baumeister and coworkers [22], Martin and coworkers [23], and Shi and coworkers [24]. These studies revealed that the association of ubiquitin aldehyde with Usp14/Ubp6 induces marked conformational changes in the 19S complex that are characteristic of the activated state. The central channel in the ATPase ring becomes enlarged, and aligned with the 20S gate, which is in its open conformation. These adaptions must account for the accelerated hydrolysis of small peptides. In addition, the catalytic domain of Ubp6 becomes located closer to Rpn11, a critical deubiquitinating enzyme, which is located above the ATPase ring. Rpn11 must remove the ubiquitin chain from the polypeptide if it is to be translocated by the ATPases into the core 20S particle.

The finding that Usp14 functions in proteasome activation was quite surprising because Usp14/Ubp6 had been shown by Finley’s group to inhibit 26S proteasome activity both allosterically [6] and also by functioning as a timing device that, by removing the ubiquitin chains, limits the time that a substrate resides on the proteasome [25]. Thus, Usp14/Ubp6 enzymatic activity can lead to the release of the protein substrate without its degradation [25,26]. In fact, based on this insight, small-molecule inhibitors of Usp14 were developed as potential therapeutics to enhance the clearance of certain hard-to-degrade, toxic polypeptides [26].

Our finding that Usp14 can function both as an inhibitor and an activator of proteolysis appeared contradictory and initially was quite perplexing. The simplest hypothesis to resolve these apparently conflicting roles of Usp14/Ubp6 would be that Usp14 normally inhibits proteolysis allosterically, but when it binds a ubiquitin chain on a potential substrate, Usp14 allosterically stimulates the proteasome’s degradative activities (Figure 1) [2,3,4]. Presumably, this activated state is then maintained until the ubiquitin chain is no longer bound to Usp14, as must occur when proteolysis is completed, or when the substrate is released.

Indeed, in the absence of a substrate, and independently of its role as a DUb, Usp14 was shown by Hyoung Tae Kim to inhibit multiple proteasomal processes [4]. The 26S proteasomes purified from MEF cells lacking Usp14 (Usp14KO 26S) consistently exhibited greater basal and ATPγS-stimulated activities of all three peptidase sites than did WT 26S [4]. Accordingly, the addition of a catalytically dead Usp14 (due to mutation of the active site cysteine) repressed allosterically those three peptidase activities. Because Usp14 suppresses all three activities simultaneously, most likely, it inhibits the substrate entry step rather than altering the catalytic activities of the three peptidase sites. In addition, the Usp14KO proteasome showed higher Rpn11 activity (e.g., they disassemble tetra-ubiquitin chains faster) than WT [4]. Since the catalytic domain of Usp14 can sterically hinder substrate access to Rpn11 [23], this increased Rpn11 activity in the Usp14KO 26S is probably because Rpn11’s active site becomes more accessible to ubiquitin chains.

Usp14KO proteasomes also have a higher basal ATPase activity than WT 26S, and surprisingly, these proteasomes do not require ubiquitin chain binding to stimulate ATP hydrolysis. By itself, an unfolded protein can stimulate ATP hydrolysis maximally [4], which wild-type 26S do only when they bind both an unfolded protein and a ubiquitin chain (or ubiquitin aldehyde) [3]. In this respect, the ATPases in the Usp14KO proteasomes behave like the homologous, AAA family of ATP-dependent proteases from bacteria (Lon, ClpAP, ClpXP, and HslUV) and the archaeal proteasome regulatory complex PAN. These hexameric enzymes are all ATPases that are activated by protein substrates [27,28]. During the early evolution of eukaryotes, ubiquitin conjugation became linked to proteolysis, which must have provided much greater selectivity and regulatory possibilities for protein degradation, but it necessitated evolution of the 26S proteasome. Through the association with Usp14, the proteasome acquired the dependence on ubiquitin chain binding to stimulate substrate entry and ATP hydrolysis.

Notably, Usp14KO 26S, unlike the WT, can degrade efficiently certain unstructured proteins without ubiquitination, which probably is a result of their faster translocation into the 20S particle and greater ATP hydrolysis. Moreover, 26S proteasomes lacking Usp14 are not just an informative laboratory construct, but also must be of physiological importance since Usp14 is present normally on only a minor fraction of cell proteasomes and exists freely in the cytosol. Interestingly, Chueh-Ling Kuo in our lab has shown that the presence of ubiquitinated proteins promotes the binding of free Usp14 to the proteasome, from which it dissociates when the ubiquitinated substrates are hydrolyzed [29]. Thus, the association of 26S particles with Usp14 is a regulated step that must facilitate the degradation of ubiquitin conjugates as it links deubiquitination of the substrate with its hydrolysis.

The suppression of proteasomal ATP hydrolysis by Usp14 may also be important in preventing ubiquitin-independent proteolysis in vivo. These findings together provide a clear rationale for these bidirectional effects of Usp14 on proteasome function. In the absence of a ubiquitin-conjugated substrate, Usp14 can help to prevent wasteful ATP consumption and the non-selective degradation of non-ubiquitinated proteins by the proteasome [4]. The activation of the proteasome upon binding a ubiquitin conjugate thus enhances the selectivity of the 26S for ubiquitinated substrates, especially those with unfolded domains (Figure 1). In addition to these allosteric effects, Usp14’s DUb activity can deubiquitinate and release proteins which cannot be efficiently degraded [25].

## 3. Allosteric Activation of Proteasomal Degradation by Usp14’s UBL Domain

Because of the importance of Usp14’s UBL domain for its association with the proteasome and for the resulting stimulation of Usp14’s deubiquitinating activity [30], we hypothesized that its UBL domain might also be essential in mediating the activation of the proteasome upon substrate binding to Usp14 [5]. In fact, we found that Usp14’s UBL domain by itself allosterically stimulates the same proteasome activities as are inhibited by Usp14 in the absence of a substrate and that are activated when a ubiquitin chain binds to Usp14 (Figure 1) [5]. In contrast to the association of Usp14, with the proteasome, the binding of its UBL domain alone increases coordinately the proteasome‘s three peptidase activities, which strongly suggests enhanced gate opening into the 20S particle. Gate opening and substrate entry into the 20S particle is believed to be stimulated maximally by ATPγS. However, the UBL domain was found to stimulate peptide hydrolysis most strongly in the presence of ATPγS. In addition, Hyoung Tae Kim found that the UBL domain alone, like ATPγS, stimulates the deubiquitinating activity of Rpn11 and does so even in the presence of ATPγS [5]. These findings strongly suggested that ATPγS and the UBL domain activate the proteasome by distinct mechanisms, as discussed below.

When Usp14’s catalytic domain binds a ubiquitin chain, Usp14 can sterically inhibit Rpn11’s deubiquitinating activity. The finding that the UBL domain alone stimulates Rpn11’s activity against ubiquitin chains suggests an unexpected coordination between the actions of these two proteasomal DUbs. Furthermore, with casein, a protein with an unfolded domain, present, the UBL domain alone was found to stimulate ATP hydrolysis by the proteasomes. Because the UBL, by itself, alters the activity of the ATPases in a similar manner as does the binding of a ubiquitin chain or ubiquitin aldehyde to Usp14 [5], it is very likely that the proteasome activation upon substrate binding to Usp14 is mediated by its UBL domain.

Additional strong evidence for this conclusion was that the incubation of 26S proteasomes with Usp14’s UBL domain alone increased the degradation of ubiquitinated substrates (Ub_5_-DHFR and the ubiquitinated Sic1). Additionally, the isolated UBL domains derived from Ubiquilin1 and Rad23B also activated proteasomes, suggesting that this is a general property of such domains. Moreover, when a UBL domain was expressed in HeLa cells as the fusion EGFP-UBL, there was a clear enhancement of overall cellular proteolysis that was accompanied by a fall in the cellular level of ubiquitin conjugates [5]. This result was particularly striking, because the overexpression of UBL would be expected to compete with the binding of substrates and shuttling factors to the proteasome and to inhibit proteolysis. In fact, we did observe such an inhibition with higher levels of UBL expression. This stimulation of proteolysis by the UBL domain in cells thus must result from proteasome activation in accord with the results with purified 26S proteasomes.

## 4. Proteasome Activation by the UBL-Domain-Containing Proteins

**Figure 2:**
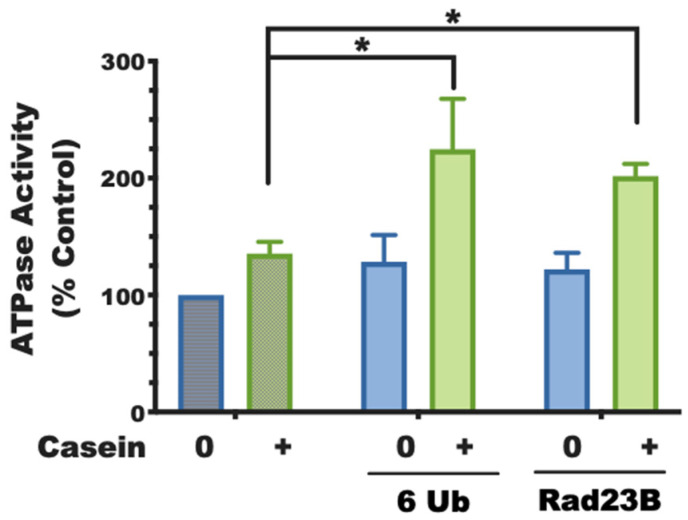
Activation of the 26S ATPases requires two signals. The basal rate of ATP hydrolysis by rabbit muscle proteasomes can be stimulated by a ubiquitinated substrate provided it contains both a ubiquitin chain (e.g., here, a linear hexa-ubiquitin, 6 Ub) and a protein with a loosely folded domain (e.g., casein). Normally, these two features are covalently linked in a ubiquitinated substrate but, as shown here, they can activate if on separate molecules. Additionally, isolated UBL domains or a full-length UBL protein (e.g., Rad23B) can replace ubiquitin chains in activating the 26S ATPases (adapted from [8]). (Mean of three experiments ± SEM; * denotes *p* < 0.05).

As discussed above, when Usp14 has bound a substrate and is in its catalytically active conformation, its UBL domain somehow alters its interactions within the 19S particle, leading to proteasome activation [5]. As a result, the substrate entry channel in the ATPase ring and the gated channel into the 20S open, allowing more rapid entry and hydrolysis of peptides to occur [8]. If there is an unfolded region on the ubiquitinated protein, there is an additional activation step which causes the ATPase activity to rise (Figure 2) [8,31]. These substrate-dependent structural changes increase the capacity of the 26S complex to degrade selectively and efficiently ubiquitinated proteins when they appear.

The UBL domain is defined as an arrangement of five beta-sheets around a helical core, called a beta-grasp, and it is a fairly common protein motif [32]. Unlike the structurally related ubiquitin family of small proteins (Ubiquitin, ISG15, Nedd8, FAT10, URM1, UFM1, and SUMO), UBL-domain-containing proteins do not become conjugated to other proteins. Instead, these UBL-domain-containing proteins exhibit a variety of enzymatic activities, many of which probably also contribute to protein degradation as they bind to proteasomes. The affinities of the UBL domain proteins for proteasomes are high but span a broad range from around 20 nM to 300 nM. In the human genome, there are at least 60 genes encoding proteins with such a UBL domain, and nearly half of these are known to bind to proteasomes. The UBL domain is essential for the functioning of the several UBL-UBA shuttling factors, which deliver ubiquitinated substrates to the proteasomes, including Rad23b and Rad23a, Ubqln1-4, or Ddi2, [33,34,35]. In addition to Usp14, several proteasome-associated DUbs, including Usp4 [36] and Usp7 [37], contain one or even multiple UBL domains. Certain ubiquitin-ligases, such as Parkin [38] and FBXO7 [39], and the nuclear phosphatase, UBLCP1 [40], also contain UBL domains that allow them to bind to certain 26S proteasomes.

**Figure 3:**
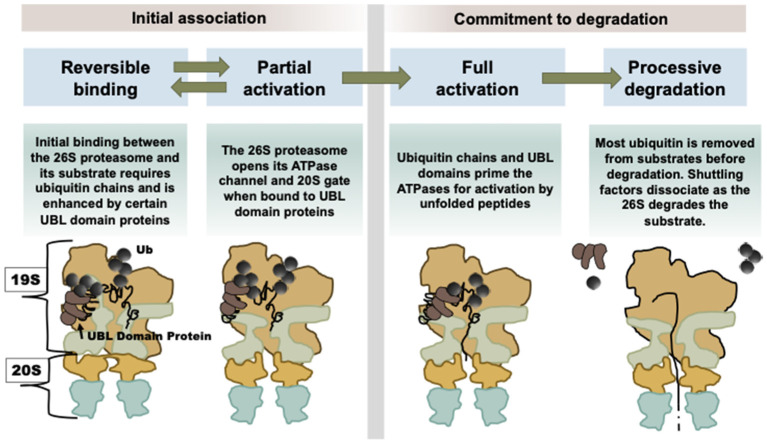
Our present understanding of how UBL-UBA shuttling factors stimulate proteasomal degradation of client proteins. This mechanism summarizes multiple steps leading to processive degradation. Shuttling factors (Rad23A, Rad23B, Ddi2, Ubqln1, Ubqln2, and Ubqln4) facilitate the delivery of ubiquitinated proteins to proteasomes and, with their UBL domains, trigger this multistep activation mechanism, which involves stimulating the ATPases (the light green objects in the 19S particle) and opening the gates of the 20S α-subunits (the orange objects in the 20S particle) (for further discussion, see [31]).

Based on our findings with Usp14, we have studied eight of these UBL-domain-containing proteins [8] to test for their ability to activate proteasomes using two assays: (1) the capacity to enhance gate-opening and peptide entry into the 20S; (2) the additional activation of 26S ATPases, which also requires the presence of a loosely folded protein [29]. Both the isolated UBL domains and the full-length UBL proteins containing them could consistently replace the requirement for a ubiquitin chain for 26S activation, including the 2-3-fold stimulation of ATP hydrolysis, which requires both the UBL domain and an unfolded protein (experimentally supplied as casein in trans to the purified proteasomes). Unlike ubiquitin itself, which must be incorporated into chains or attached at multiple sites to substrates to activate proteasomes, a single UBL domain is sufficient to activate proteasomes (Figure 3).

In Usp7, which contains five UBL domains, the number of ubiquitin domains influences the affinity for proteasomes but does not increase further the extent of activation. This activation depends upon the association of the UBL with the 19S component, Rpn1 (see below). Interestingly, when UBL domains are incubated with free 20S proteasomes, they inhibit rather than stimulate peptide hydrolysis (Collins and Goldberg, unpublished).

We and many others had assumed that 26S proteasomes in the presence of ATPγS show a maximal increase in peptidase activity through the conformational alignment of the ATPase ring and opening of the 20S gate [41,42,43]. Therefore, it was a surprise to find that this ability of the UBL domains to increase peptide hydrolysis and the increase in activity upon ATPγS binding were additive in increasing peptide hydrolysis. In fact, the activation by the UBL domain proteins (such as the substrate-bound form of Usp14) was most easily observed with ATPγS in the buffer [8]. Because a further gate-opening in the 20S seemed quite unlikely on structural grounds, activation by the non-hydrolysable ATP analog and a UBL domain protein must involve distinct mechanisms. By using covalent fluorescent probes of the 20S active sites, we determined that the UBL-domain-containing proteins were not increasing the rate of labeling of previously active proteasomes (i.e., they did not open the gate wider or faster). Instead, they were increasing the number of proteasomes labeled, thus indicating that the UBL domain proteins mobilize a subset of proteasomes that were not activated by nucleotide binding. This unexpected result is consistent with sub-classifications of 26S proteasome structures in cells obtained by the Baumeister lab using cryo-EM tomography, which showed that a large number of intracellular 26S (around 70 percent) are normally in an inactive state [41,43]. In these cases, a remarkable number of proteasomes have closed gates despite the presence of high levels of ATP in the cell, but presumably become active upon binding a UBL domain protein.

It should also be noted that the various activators of the 26S seem to bind in distinct fashions. The UBL domain of Ubp6, the yeast homolog of Usp14, binds principally to Rpn1 at a region called T2 [44]. Rad23 also binds Rpn1, but in a distinct region called T1 [44]. However, Rad23B can also associate with proteasomes through Rpn10 and Rpn13 [45]. UBLCP1 binds Rpn1 [46], and Parkin probably binds through Rpn13 [47] although Rpn10 has also been a proposed as a Parkin-binding site [48]. Even a subunit not known for ubiquitin binding, Rpn6, seems to be bound by Usp4’s UBL domain [36]. Given the diversity of binding sites, and possible activation sites, it seems very likely that there are multiple pathways for ubiquitinated substrates to associate with proteasomes in vivo and activate proteolysis.

It therefore seems likely that the fate of a ubiquitinated substrate may differ if it binds a proteasome directly through one of the known ubiquitin receptors (Rpn10, Rpn13, or Rpn1) and then to Usp14, or if it is delivered by a UBL-UBA protein to a 26S proteasome. For different ubiquitinated substrates, these different modes of binding to the 26S may not activate or may not lead to the capture of a loosely folded domain of the protein by the ATPase [31]. In addition, the shuttling UBL-UBA factors are capable of activating a group of otherwise “somnolent” (latent) proteasomes, while also protecting the substrate from premature deubiquitination and release [49]. In the future, it will be of major importance to compare structurally and kinetically the degradation of a ubiquitinated substrate that binds directly to a 26S proteasome versus a proteasome that is activated by its association with Parkin or another UBL protein.

These observations clearly indicate that there is greater heterogeneity and complexity in the mechanisms for proteasomal degradation in the cell than we had thought previously. Such heterogeneous activation mechanisms are probably needed to handle the great diversity of proteins that are being continually degraded in cells. By contrast, the majority of published studies of proteolysis by isolated 26S proteasomes have utilized simple, relatively homogeneous, and easily degraded ubiquitinated substrates, and thus probably have missed the actual challenges that the 26S proteasomes encounters within a cell in degrading diverse types of cell proteins.

## 5. ZFAND5, an Activator of the Proteasome and Protein Degradation in Muscle

We have also studied a very different type of proteasome activator, ZFAND5/ZNF216. This protein is normally expressed at high levels in the brain and heart [8], but its roles in these tissues is not clear. Normally, its levels in the skeletal muscle and liver are very low, but its expression in muscle rises in a variety of catabolic conditions, such as fasting and denervation, which lead to muscle atrophy [11]. Most importantly, muscles lacking ZFAND5 have a reduced capacity to undergo atrophy [11]. Accordingly, the content of ZFAND5 increases in cultured myotubes upon treatment with dexamethasone, which is a useful cellular model of atrophy [10,11]. These conditions all cause a rapid increase in overall protein degradation in muscle by the ubiquitin–proteasome pathway [50]. Thus, ZFAND5 behaves like one of the 100–200 atrophy-related genes which are induced in various catabolic-related conditions and promote muscle wasting [51]. These observations suggested that ZFAND5 plays a key role in the activation of muscle proteolysis. Furthermore, Hishiya et al. also showed that ZFAND5 associates with both ubiquitin conjugates and also with proteasomes, strongly suggesting a direct role in regulating proteasomal degradation [11].

**Figure 4:**
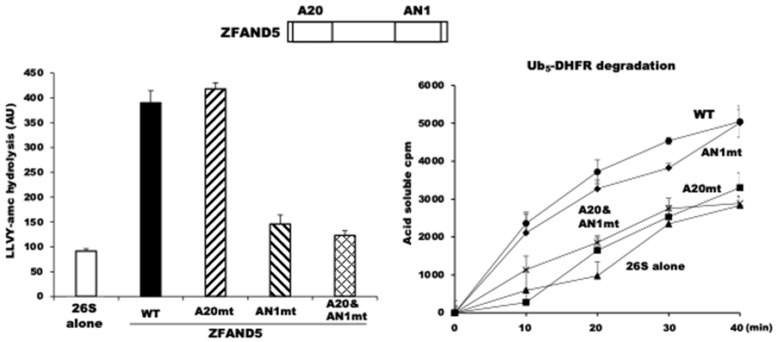
ZFAND5 directly activates purified 26S proteasomes and increases their ability to hydrolyze peptides and ubiquitin conjugates. (Left Panel) ZFAND5 stimulates the peptidase activity of 26S proteasomes. This activation is dependent on ZFAND5’s C-terminal AN1 domain. (Right Panel) ZFAND5 enhances also degradation of ubiquitinated ^32^P-dehydrofolate reductase. Its A20 domain is essential for the increased degradation of ubiquitinated proteins.

Accordingly, we found that in MEF cells that lack ZFAND5, the degradation of long-lived cell proteins, the bulk of cell proteins, is significantly less than in WT MEF cells, and that the addition of ZFAND5 to normal cell extracts could stimulate the proteasomal degradation of endogenous proteins [10]. To understand the enzymological role(s) of ZFAND5, Donghoon Lee studied the effects of recombinant ZFAND5 produced in *E. coli* on the activities of affinity-purified 26S proteasomes. Pure ZFAND5 markedly stimulates the three peptidase activities of the proteasomes (Figure 4), which strongly suggests that ZFAND5, like other proteasome activators, triggers the alignment of the central channel in the ATPase ring with the entry channel into the 20S and gate-opening in the 20S particle [10].

Purified ZFAND5 also increased the ATPase activity of the 26S proteasome and its ability to degrade ubiquitinated proteins (Figure 4). Thus, it must be altering the functioning of the 19S regulatory complex. By contrast, ZFAND5 caused no such stimulation of the activity of purified 20S proteasomes. On the contrary, ZFAND5 actually inhibits the degradation of certain protein substrates by free 20S particles.

Exactly how ZFAND5 enhances proteasomal activity is an important question that we are intensely studying. When ZFAND5 and 26S proteasomes were resolved on native PAGE, the migration of both doubly and singly capped proteasomes was reduced [10]. Moreover, free 19S migrated more slowly when incubated with ZFAND5. By contrast, the migration of 20S was not altered by ZFAND5. Thus, ZFAND5 appears to bind to the 19S and to induce a large conformational change in its structure. Unpublished single particle analysis in collaboration with Louis Colson and Ying Lu (Harvard Medical School) clearly demonstrates that ZFAND5 promotes the binding of ubiquitinated substrates to the proteasome. Additionally, chemical cross-linking experiments in collaboration with Lan Huang and Xiaorong Wang (University of California, Irvine) and cryo-EM analysis in collaboration with Ying Lu and Yanan Zhu (Oxford University) together indicate that ZFAND5 interacts with specific 19S subunits, including Rpt5, but how these interactions enhance proteasomal activity remains uncertain.

Unlike the five other members of the ZFAND family, which each contain the AN1 Zn finger domain, ZFAND5 contains a second Zn finger domain, A20, at its N-terminus. Prior NMR studies and our site-directed mutations that inactivated each Zn finger domain demonstrated that the A20 Zn finger binds ubiquitin, and ubiquitin-binding domains resembling A20 have been described in other proteins [52]. However, the role of the AN1 Zn finger remains unclear, even though it is the structural domain that defines the ZFAND family. Mutations of two cysteines in the AN1 domain of ZFAND5 abolished its ability to stimulate the proteasome’s peptidase activity (Figure 4). By contrast, ZFAND5’s ability to stimulate the hydrolysis of a ubiquitinated protein requires its A20 domain, presumably to bind the ubiquitin chains. These experiments with pure components appear relevant to the ZFAND5 function in regulating proteolysis in cells, since the addition of ZFAND5 to radiolabeled cell extracts increased the degradation of endogenous cell proteins, and this required its A20 domain and thus, presumably, binding of the ubiquitin chain to ZFAND5.

The ability of ZFAND5 to stimulate the activities of the 26S complex raises many intriguing mechanistic and physiological questions, such as: Why is ZFAND5 important specifically in muscle atrophy? Is it simply accelerating proteasome function? Its physiological roles in other cells remain an important topic for further study. Because ZFAND5 interacts with both ubiquitin and proteasomes and accelerates the hydrolysis of ubiquitinated proteins, ZFAND5 may serve on the proteasome as an additional binding site for ubiquitinated substrates, or it may function in an analogous manner to a shuttling factor (such as Rad23B) to deliver certain ubiquitin conjugates to the proteasome and activate its degradative capacity. However, its structure differs completely from the UBL-UBA shuttling factors and from the proteasome’s known ubiquitin receptors. Moreover, it remains unclear how or why the acceleration of protein degradation in atrophying muscles would depend on such an additional “shuttling factor”.

Furthermore, unlike the standard cellular shuttling factors that are present in all cells, ZFAND5 is not a very stable cell constituent. Even in muscle, it is rapidly degraded (e.g., especially upon heat shock). In other cells, it is induced by several inflammatory mediators [53]. Interestingly, after exposure to proinflammatory cytokines (TNF-α and IFN-γ), the most abundant peptides being degraded by proteasomes are derived from ZFAND5 [54]. Thus, in many cells, ZFAND5 appears to be both rapidly induced and then rapidly destroyed. However, its high levels in the heart and brain suggest that ZFAND5 is more stable and plays distinct roles in proteostasis in those organs. Although ZFAND5 is clearly a novel type of proteasome activator that helps to stimulate proteolysis by the UPS in atrophying muscles when degradation rates are most rapid, its mechanisms in stimulating proteasomal activity, as well as its physiological role in non-muscle tissues, are important topics for future work to unravel.

## 6. Activation of Proteasomes and Protein Degradation by cAMP and PKA

A large number of post-synthetic modifications of 26S proteasome subunits have been reported, especially phosphorylation [13], which has been proposed to influence the localization [55], activity [56], and formation [57,58] of the 26S proteasome. Phosphorylation of one of the 19S ATPases, Rpt6, in neurons by Ca^2+^/calmodulin-dependent protein kinase II (CaMKII) has been reported to cause proteasome entry into dendrites and promote synaptic plasticity [55,59]. Moreover, phosphorylation of Rpt6 by cAMP-dependent proteins kinase (PKA) was reported to increase proteasome activity against small peptides [56,60,61]. However, the effects of these modifications on the proteasome’s capacity to degrade ubiquitin conjugates and on protein degradation in cells were not examined. We therefore decided to systematically investigate the possible effects of cAMP and PKA on proteasome function and protein degradation by the UPS [15]. Our studies led to several surprising and novel insights—most importantly, that PKA phosphorylates the 19S subunit Rpn6 (and not Rpt6) and thus stimulates several key proteasomal processes, and that PKA activation also enhances the cell’s capacity to degrade short-lived ubiquitinated proteins, including various aggregation-prone proteins that cause major neurodegenerative diseases.

**Figure 5:**
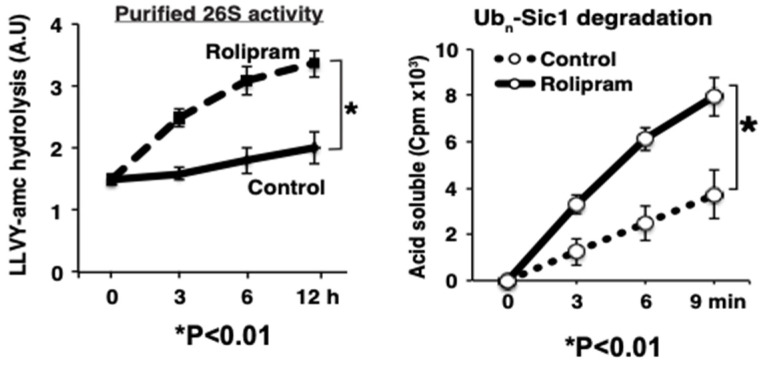
26S proteasomes from mouse myotubes treated with the PDE4 inhibitor, rolipram, are more active in hydrolyzing peptides and a ubiquitinated protein. C2Cl2 myotubes were incubated with rolipram, and, at different times, samples were taken and 26S proteasomes purified by the UBL method (Left Panel). Chymotrypsin-like peptidase activity was increased in the treated cells. (Right Panel) After 6 h treatment of cells with rolipram, the purified proteasomes show a greater capacity to degrade ubiquitinated ^32^P-Sic1 (adapted from [15]). (Mean of three experiments ± SEM; * denotes *p* < 0.01).

These exciting conclusions are based on a large variety of pharmacological and biochemical findings [37]. (1) The activation of adenylate cyclase with forskolin stimulated within minutes PKA and proteasomal activity. (2) 26S proteasomes were also more active upon treatment with rolipram, an inhibitor of PDE4, the enzyme that hydrolyzes and inactivates cAMP (Figure 5). (3) Forskolin caused a similar activation of proteasomes in multiple cell lines, including HEK293A, neuroblastoma, myotubes, and cardiomyocytes, and in collaborative studies, we found a stimulation of proteasome activity in the brain upon treatment of mice [18] or zebrafish [16] with rolipram. (4) These stimulatory effects on the proteasome could be blocked by the PKA inhibitor, H89. (5) Enhanced peptidase activity was observed also after purified 26S proteasomes were treated with recombinant PKA. (6) Conversely, this enhancement of proteasome activities was reversed by phosphatase treatment with protein phosphatase. (7) Incorporation of the overexpressed phosphomi-metic mutant (S14D) of Rpn6 into the proteasome also enhanced peptidase activity. (8) After rolipram treatment, PKA’s catalytic subunit was found in association with the 26S particles.

To study the possible effects of cAMP and proteasome activation on protein breakdown in cells, we used radioactive amino acids to label different fractions of cell proteins [62] and made the important, unexpected finding that raising cAMP levels in cells promotes the breakdown of short-lived proteins but does not affect the degradation of the bulk of cell proteins [15]. In addition, forskolin treatment enhanced the rapid degradation of several short-lived model UPS substrates, including GFP fusions ubiquitinated via the CL1 degron, the N-end rule pathway, and the UFD pathway. Although these substrates are ubiquitinated at distinct rates by different ubiquitin ligases, their degradation was enhanced similarly. Since these long-lived cell components are also degraded primarily by the UPS [63,64], it is unclear how PKA selectively enhanced the proteasomal degradation of these short-lived proteins without affecting the degradation of the bulk of cell proteins. Possibly, for these long-lived components, ubiquitination rather than proteasome function is the rate-limiting step in their degradation. On the other hand, these experiments indicated that cAMP via PKA also increases in minutes the total levels of ubiquitinated proteins in the cells. Therefore, it remains possible that PKA promotes the selective ubiquitination of certain short-lived cell proteins as well as their enhanced degradation by proteasomes.

The treatments that raised cAMP in cells and stimulated proteolysis enhanced the capacity of their 26S proteasomes to degrade model ubiquitinated proteins (Ub_n_-Sic1 and Ub_5_-DHFR). Degradation of ubiquitin conjugates involves many ATP-dependent steps [31,65]; PKA-induced phosphorylation enhanced the two intermediate steps that we can measure, the rate of peptide entry and hydrolysis by the 20S particle and also ATP hydrolysis. Both steps are also activated upon binding of a ubiquitinated substrate, as discussed above, but the proteasome activation by the substrate was found to be additive with the activation by Rpn6 phosphorylation and presumably involves distinct mechanisms. The enhanced ATPase activity of these proteasomes is of particular interest since substrate unfolding, deubiquitination, and translocation are all ATP-dependent processes [31,65], and the rate of ubiquitin conjugate breakdown is directly proportional to the rate of ATP consumption [9]. Therefore, the enhanced ATP hydrolysis probably drives the increased capacity to digest ubiquitin substrates.

While several polypeptides were phosphorylated in the proteasomal preparations, mass spectrometry and antibody analysis identified Rpn6 as the only proteasomal subunit that was consistently phosphorylated and correlated with activation [15]. In multiple studies, we could not confirm modification of the ATPase subunit Rpt6 by PKA, as had been reported previously [56]. Rpn6 appears ideally situated to influence multiple 26S functions. It is a component of the 19S lid and seems to function as a molecular clamp that interacts with both the ATPase ring and the 20S core particle [66]. The special role in Rpn6 of Serine 14 has also been supported by the mutagenesis of plasmids, specifically by the capacity of phosphomimetic mutant (S14D) to enhance and the “phospho-null” S14A mutation to decrease both proteasome activity and the clearance of aggregation-prone proteins in cells. It is noteworthy that prior studies have indicated an additional important regulatory role of Rpn6. Overexpression of WT Rpn6 was shown to enhance somehow proteasomal peptidase activities in *C. elegans* [67] and human stem cells [68] and to even enhance the longevity of *C. elegans* [67]. Perhaps these intriguing effects of Rpn6 overexpression are related to its activation by PKA and enhanced elimination of misfolded proteins or to its structural role in clamping together the 19S and 20S complexes.

## 7. cAMP Promoted Degradation of Disease-Causing Mutated Proteins

In principle, pharmacological agents that enhance proteasome function could be very valuable in combating various diseases resulting from the toxic accumulation of misfolded proteins. In the major neurodegenerative diseases (e.g., amyotrophic lateral sclerosis (ALS), Alzheimer’s, Parkinson’s, and Huntington’s disease), aggregation-prone proteins build up, often as protein inclusions that contain ubiquitin chains and proteasomes [69]. One important factor that seems to contribute to the pathogenesis of neurodegenerative diseases is the progressive impairment of the capacity of the UPS to degrade misfolded proteins. In fact, several studies of neurodegenerative disease models have suggested that proteasome function is impaired when these misfolded proteins accumulate in cells [18,69,70,71].

Our studies in cell cultures and our collaborative studies in mouse and zebrafish models indicate that raising cyclic AMP can augment the degradation of WT and mutant forms of tau, FUS, TDP43, SOD1, and huntingtin, all of which are implicated in the pathogenesis of major neurodegenerative diseases. In collaboration with Natura Myeku and Karen Duff (Columbia University), we investigated the effects of tau accumulation on proteasome function in the mouse brain in a model of tauopathy and degradation of misfolded proteins by the UPS via a UPS reporter mouse line expressing Ub-G76V-GFP [18]. The gradual accumulation of insoluble mutant tau was associated with a decrease in the peptidase activity of brain 26S proteasomes, higher levels of ubiquitinated proteins, and an accumulation of undegraded Ub-G76V-GFP. Purified 26S proteasomes from mice with the tauopathy were less active in hydrolyzing ubiquitinated proteins, small peptides, and ATP. However, administration of rolipram restored proteasome function in the mouse brains, presumably through proteasome subunit phosphorylation by PKA. This treatment of the mice decreased levels of aggregated tau and even improved their cognitive performance [18]. More recently, Myeku has confirmed these findings with other treatments that raise cAMP [72]. Interestingly, in cell culture and in the mouse tauopathy model, raising cAMP decreased the content of mutated tau and these other aggregation-prone proteins in both soluble and insoluble (i.e., aggregated) fractions by a proteasomal process and not through autophagy. Because the capacity of proteasomes to digest large protein aggregates is limited, the rapid decrease in these protein aggregates probably occurred through accelerated hydrolysis of soluble tau or micro-aggregates of tau before the mutated species formed large aggregates [16].

It is also noteworthy that rolipram treatment of the transgenic mice accelerated the clearance of mutant tau only in the early stages of the disease, where raising cAMP not only enhanced proteasome activity in the brain and promoted the clearance of ubiquitinated proteins and hyperphosphorylated tau [18]. In these animals, the gradual accumulation of mutant tau correlated with the progressive decline in proteasomal capacity for peptide and ubiquitin conjugate degradation. In other words, the same processes that decreased with disease progression were stimulated by PKA in vitro and were reversed by rolipram in vivo. More recently, in collaboration with David Rubinsztein and coworkers (Cambridge Institute of Medical Research), using zebrafish models of Alzheimer’s and Huntington’s disease, we showed that rolipram also promotes the clearance of two different disease-associated tau mutants and a mutant huntingtin exon 1 containing a 71-polyglutamine expansion [16]. Furthermore, rolipram treatment decreased the associated neuronal death as well as the developmental abnormalities resulting from overexpression of these aggregation-prone proteins. Interestingly, agents that raise cAMP have also been reported to improve memory in humans, including Alzheimer’s disease patients [73,74]. These findings together certainly raise the possibility that such agents may also be useful to help to clear the toxic proteins and thus slow the progression of neurodegenerative diseases.

## 8. Hormones That Raise cAMP Stimulate Proteasomes and Protein Degradation

**Figure 6:**
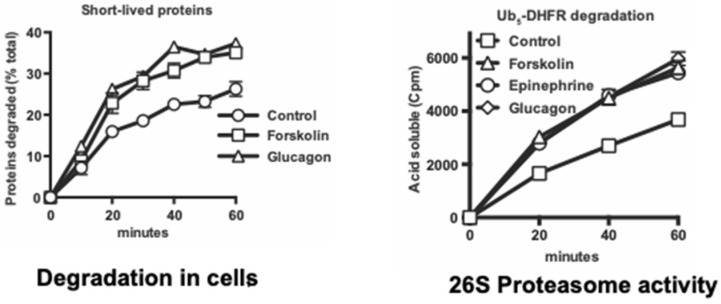
In mouse hepatocytes, glucagon and epinephrine, like forskolin, rapidly stimulate breakdown of short-lived proteins and the proteasomes’ ability to degrade ubiquitinated substrates. (Left Panel) Short-lived proteins in primary mouse hepatocytes were initially labeled for 20 min with ^3^H-phenylanine, as described previously [62,64]. After labeling and re-suspension in chase medium, degradation was measured in the presence of glucagon or forskolin. (Right Panel) 26S proteasomes were purified by the UBL method from (nonlabeled) mouse hepatocytes treated for 1 h with either forskolin, epinephrine, glucagon, or the vehicle. These treatments increased the ability of proteasomes to degrade the model UPS substrate, ubiquitinated ^32^P-labeled dihydrofolate reductase (adapted from [16]).

Because cAMP and PKA mediate many diverse hormonal responses and physiological responses, we also investigated whether a similar proteasome activation occurs in vivo in various physiological conditions that raise cAMP. The first cAMP-mediated metabolic response discovered by Sutherland and coworkers was the stimulation of hepatic glycogen breakdown by epinephrine (e.g., as occurs in exercise) and glucagon (e.g., as occurs in fasting). We found that treatment of mouse hepatocytes with epinephrine or glucagon also stimulated Rpn6 phosphorylation and the 26S proteasomes’ capacity to degrade ubiquitinated proteins and peptides in a very similar fashion to forskolin (Figure 6) [17].

Moreover, like forskolin, these hormones promoted the selective degradation in the hepatocytes of short-lived proteins, which include misfolded and regulatory proteins, but not the bulk of cell proteins. In additional experiments, proteasome activities and Rpn6 phosphorylation were also shown to increase similarly in working perfused rat hearts following epinephrine treatment, and also in leg muscle biopsies from exercising human volunteers, and in electrically stimulated rat hindlimb muscles [16]. Furthermore, in wild-type mouse kidney cells, but not in cells lacking PKA, treatment with the antidiuretic hormone, vasopressin, stimulated within 5 min proteasomal activity, Rpn6 phosphorylation, and the selective degradation of short-lived cell proteins. In livers and skeletal muscles of mice fasted overnight, cAMP levels, Rpn6 phosphorylation, and proteasomal-specific activities increased without any change in proteasomal content [16].

These observations in diverse cells and tissues clearly demonstrated that proteasome activation and enhanced degradation of short-lived proteins are physiological responses to cAMP and not just pharmacological artifacts. These findings also confirmed our earlier conclusion that Rpn6-S14 is a bona fide PKA target [15] and that it is phosphorylated in vivo under very different physiological conditions [17]. In our prior study [15], and in mass spectrometry studies of exercising human muscles [75], there was no change in the phosphorylation of the ATPase subunit Rpt6, which had been initially reported to be modified by PKA [56], by CaMKII [76], and by PKG [77,78].

Proteasomal activity and the ability to degrade short-lived cell proteins thus appear to rise together with the increased glycogenolysis and triglyceride breakdown that are induced in the liver, muscle, and heart by epinephrine via cAMP during the fight-or-flight response and in the liver by glucagon in fasting. In tissues of fasted mice, even up to 2 days, when degradation rates are very high, proteasomal activities increased without any changes in 26S content. There has been appreciable controversy about how starvation affects proteasome content in cultured mammalian cells. Proteasome levels have been reported to rise very rapidly upon nutrient deprivation [79], to fall slowly due to accelerated autophagy [80], or to remain unchanged for many hours [64]. Whatever the basis for these divergent results in cultured cells, the physiological mechanism to increase the capacity for protein degradation in fasting in vivo is through post-synthetic modification of proteasomes, not through the production of new proteasomes [17].

This increased proteasome activity in mouse muscles and liver was clearly evident by 12 h after food was removed from fed animals and thus represents a rather rapid metabolic response to food deprivation. This timing suggests that a similar enhancement of proteolysis should also occur in humans in these tissues after an overnight fast (i.e., between dinner and breakfast). Incidentally, this rapid response long precedes the FoxO-mediated induction of ubiquitin ligases and autophagy genes that leads to muscle wasting, especially the breakdown of myofibrils, which is evident in rat and mouse muscles at 1–2 days after food deprivation [81]. Moreover, the FoxO-mediated response stimulates the breakdown of long-lived proteins, the great bulk of cell proteins, to provide the starving organism with amino acids for gluconeogenesis and energy production and thus serves distinct physiological functions from the PKA-mediated enhancement of the degradation of short-lived proteins.

## 9. Activation of Proteasomes and Protein Degradation by cGMP and PKG

Because of these exciting discoveries about cAMP and PKA and especially their ability to enhance the cell’s capacity to degrade disease-associated, mutant proteins, we decided to investigate whether other signal transduction systems had similar effects and focused on cGMP and protein kinase G (PKG) [16]. While cAMP serves as a second messenger for multiple neurotransmitters and peptide hormones, cGMP functions as an intracellular second messenger that is synthesized by soluble guanylyl cyclases in response to NO, certain peptide hormones (e.g., natriuretic peptides), and certain cholinergic agents. Most interest in cGMP has focused on its mediating peripheral smooth muscle relaxation, which has major medical applications. For example, the inhibitors of the cGMP-specific phosphodiesterase 5 (PDE5), sildenafil or tadalafil, are widely used to treat erectile dysfunction and pulmonary hypertension, while stimulators of cGMP synthesis (e.g., Riociguat) are used clinically to treat cardiac failure. While other protein kinases may also activate the UPS and may also have therapeutic applications against neurodegenerative diseases, we have focused on the new actions of cAMP and cGMP because of the extensive knowledge already available about their pharmacology and physiology. Although raising cAMP globally has been shown to have untoward effects (e.g., nausea and emesis) that have prevented its usage in patients, treatments that raise cGMP do not elicit such effects, can enhance cerebral blood flow, and can also promote memory in human [82].

The ability of cGMP and PKG to alter protein turnover generally or to combat the progression of neurodegenerative disease had not been studied systematically. In a mouse model of a cardiomyopathy caused by overexpression of mutant αβ crystallin, Ranek et al. [78] showed that treatment with sildenafil to raise cGMP could increase proteasomal peptidase activity in the heart and reduce the levels of mutant αβ-crystallin and cardiac hypertrophy, thus indicating that raising cGMP may have therapeutic potential in treating hereditary cardiomyopathies [83]. However, it was not clear how cGMP altered proteasome activity, whether these intriguing effects were specific to the heart or if cGMP may enhance breakdown of intracellular proteins generally, and whether it may also influence protein ubiquitination or autophagy. We therefore investigated these important questions.

**Figure 7:**
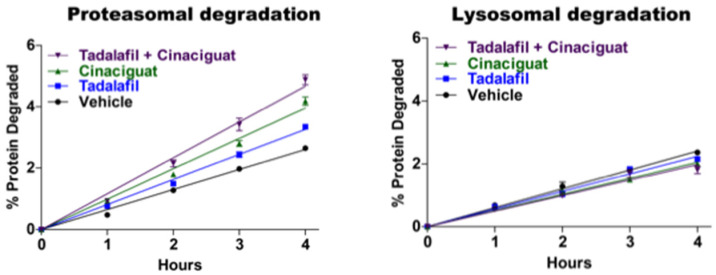
Agents that raise cGMP increase the degradation of proteins by the UPS, but not by autophagy. Proteins in SY5Y cells were prelabeled with ^3^H-phenylanine for 20 h to label long-lived cell proteins as described previously [62,64]. Proteasomal degradation reflects the net decrease in total protein breakdown in the presence of the proteasome inhibitor bortezomib. Lysosomal degradation represents the net decrease in the presence of the inhibitor of lysosome acidification concanamycin A (adapted from [16]).

Extensive studies in our lab by Jordan VerPlank demonstrated that cGMP and PKG also stimulate multiple proteasome activities and cellular proteolysis by the UPS without any effect on autophagy (Figure 7).

The experiments implicating cGMP and PKG in proteasome regulation [16] resembled our earlier studies with cAMP and PKA. (1) Treating SY5Y neuroblastoma cells as well as several other cell lines with either of two inhibitors of phosphodiesterase 5, which hydrolyzes cGMP selectively, or with a stimulator of soluble guanylyl cyclase (Riociguat or BAY41-2722) rapidly enhanced proteasome peptidase activity. (2) After affinity purification from the treated cells, the 26S proteasomes hydrolyzed peptides by the chymotrypsin-, trypsin-, and caspase-like sites 2–3-fold faster, consumed ATP faster, and showed an enhanced capacity to degrade a model ubiquitinated substrate. These findings clearly indicated activation of the 19S Regulatory Particle, whose ATPase activity drives ubiquitin conjugate degradation and substrate entry into the core 20S particle. (3) These effects of cGMP could be blocked by inhibitors of PKG and (4) appear physiologically relevant, since similar findings were obtained when we used the cholinergic agonist carbachol to raise cGMP. (5) Proteasome activation resulted from direct phosphorylation by PKG because treatment of purified 26S proteasomes with recombinant PKG stimulated their peptidase activity, while (6) incubation with Lambda phosphatase reversed this activation and also reversed the activation induced in cells by sildenafil treatment. To our surprise, in stimulating purified proteasomes, or in cells, PKG did not modify Rpn6, the subunit phosphorylated by protein kinase A, or Rpt6, as had been suggested previously [56]. Despite appreciable effort and extensive mass spectrometry, we unfortunately still have not succeeded in identifying the key component of the 26S particle that is phosphorylated and causes the enhanced catalytic activities.

Although the 26S proteasomes activated by treatments that raise cGMP behaved similarly to those from cells where cAMP levels rose, the effects on cAMP and cGMP on cellular protein degradation were quite different. Experiments with radiolabeled amino acids showed that raising cGMP, like raising cAMP, stimulated the degradation of short-lived proteins [16]. However, unlike cAMP, cGMP also markedly increased the degradation of the long-lived components (the bulk of cell proteins) without affecting lysosomal proteolysis (Figure 7). In addition to stimulating proteasome activity and cellular proteolysis within five minutes, these treatments simultaneously raised the cellular level of ubiquitinated proteins, whose levels gradually returned to baseline in the next hour or two. Because of the simultaneous enhancement of ubiquitin conjugate degradation by proteasomes, this rise in ubiquitin conjugates must underestimate the actual stimulation of protein ubiquitination by PKG.

**Figure 8:**
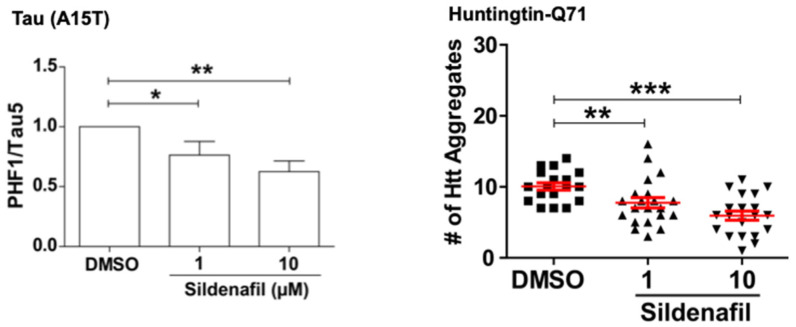
Raising cGMP levels promotes the clearance of mutant proteins in zebrafish models of a tauopathy and Huntington’s disease. (Left Panel) Zebrafish larvae expressing the tauopathy-associated A15T mutation in neurons were treated with sildenafil for 5 days. Their content of the hyperphosphorylated tau, expressed as a fraction of the total tau, was decreased, indicating selective degradation of the disease-associated species. (Right Panel) In zebrafish larvae expressing huntingtin with a 71-glutamine repeat in the retinal photoreceptor cells, sildenafil treatment for 5 days also reduced the number of huntingtin aggregates (adapted from [16]). (* denotes *p* < 0.05, ** *p* < 0.01, and *** *p* < 0.001).

Naturally, we also tested if raising cGMP, like raising cAMP, can promote the degradation of mutant proteins that cause neurodegenerative diseases [16]. Our collaborators, Prof David Rubinsztein and coworkers at Cambridge University, using their zebrafish larvae models of tauopathies and Huntington’s disease, showed that treatment of these fish with sildenafil for four days reduced the levels of the mutant tau and huntingtin. Interestingly, these effects were indistinguishable from results obtained upon treatment of the zebrafish with rolipram to raise cAMP, presumably because both PKA and PKG stimulate clearance of the misfolded toxic proteins. These findings are of particular interest because treatment of zebrafish models of Frontotemporal Dementia and Huntington’s disease with the PDE5 or the PDE4 inhibitors not only promoted the selective degradation of the disease-associated hyperphosphorylated tau mutants (A152T and P301L), as well as huntingtin containing a 71-polyQ sequence, but both treatments also reduced the associated neuronal death and the marked developmental abnormalities seen upon expression of these mutant proteins (Figure 8).

To further test if agents that raise cGMP may help to combat the progression of other neurological diseases, in collaboration with Laura Feltri and Lawrence Wrabetz (SUNY, Buffalo) in still unpublished studies, we studied a mouse model of Charcot Marie Tooth 1B, a demyelinating neuropathy caused by the expression of mutant myelin protein zero (S63del). In the sciatic nerve of the S63del mouse, 26S proteasome activities are reduced, ubiquitinated proteins accumulate, and the Unfolded Protein Response (UPR) is activated. Treatment of the mice with sildenafil for 5 days restored proteasome function and reduced levels of polyubiquitinated proteins and markers of the UPR. Remarkably, myelin thickness, decreased amyelinated axons, and improved nerve conduction were observed. Thus, through its ability to stimulate proteasome activity and intracellular protein homeostasis, pharmacological agents that raise cGMP have the potential to combat diverse neurodegenerative and other proteotoxic diseases.

## 10. Implications of These Findings

Together, these studies have led to an important, new insight into the functioning of the UPS and proteostasis. It was widely assumed that the cell’s capacity for degradation is fixed. However, these studies with cAMP clearly demonstrate that a variety of hormones that activate adenylate cyclases, but have diverse physiological roles, all stimulate proteasome activity and the selective degradation of short-lived cell proteins. Moreover, these responses are surprisingly rapid. After vasopressin addition to renal epithelial cells, proteasome activity and Rpn6-S14 phosphorylation rose maximally within 5 min and returned to control levels by 60 min [16]. Similarly, pharmacological treatments or cholinergic agents that raise cGMP also cause a very rapid activation of proteasomes, ubiquitin conjugation, and protein degradation in cells. Thus, the cell’s capacity for degradation by the UPS can change in a highly dynamic fashion that has not been widely appreciated, and rapid increases and decreases in the proteasomes’ degradative capacity through subunit phosphorylation must be occurring frequently in different tissues in vivo.

The physiological importance of this new, dynamic regulation of proteasome function remains unclear. Presumably, enhanced degradation of misfolded and damaged proteins helps cells to maintain proteostasis during stress. In addition, the accelerated breakdown of preexistent regulatory proteins induced by cAMP or cGMP can facilitate changes in cell protein composition upon transitions to new physiological conditions (e.g., with fasting). Activation of proteolysis by cAMP and cGMP was observed in many cell types and may occur in nearly all mammalian cells. However, the specific consequences of this new mechanism for regulating proteolysis in different cells remain to be determined.

Although the rate-limiting, highly selective step in proteolysis by the UPS is normally substrate ubiquitination, the finding of proteasome activation implies that the degradation of many proteins can also be regulated at the subsequent step, ubiquitin conjugate degradation by the proteasome, and that the half-lives of many proteins can change rapidly with alterations in the proteasome’s phosphorylation state. This ability of PKA, DYRK2, and PKG to enhance rates of proteolysis by increasing proteasome activity represents a new mode of regulation of protein breakdown [12,13]. While control of ubiquitination rates influences the levels of individual proteins or small groups of related proteins, control of proteasome function allows more global, coordinated regulation of the degradation of large classes of proteins.

These two mechanisms to regulate degradation by the UPS do not necessarily function independently. In fact, in addition to enhancing proteasomal activity, cAMP and cGMP stimulate within minutes the ubiquitination of some cell proteins [15,16], suggesting that these two mechanisms to increase proteolysis are activated simultaneously by PKA and PKG (although only cGMP affects the long-lived cell constituents). Possibly, the selective ubiquitination of short-lived proteins by PKA may account for their preferential destruction when cAMP levels rise. This control of proteolysis by ubiquitination and proteasomal activities seems analogous to the two levels for the control of protein production. Transcriptional regulation of gene expression allows the highly selective control of the levels of specific proteins or groups of related proteins (such as ubiquitination), while the global regulation of ribosomal translation influences the rates of the production and accumulation of large classes of cell proteins (such as regulating proteasomal function). The discovery that PKA and PKG can both stimulate this initial step in the pathway, as well as proteasomal function, are of further therapeutic importance, as well as biochemical interest, because they suggest a multistep enhancement of protein quality control by these signaling systems. These studies also indicate many fundamental questions for further study, such as which specific cell proteins are being ubiquitinated in response to cAMP and cGMP and degraded, how PKA and PKG enhance ubiquitination, and which specific enzymes are involved in accelerating this key step.

The present studies also strongly suggest that fasting, exercise, or other conditions that raise cAMP or cGMP levels might also be beneficial in promoting the clearance of potentially toxic proteins and stimulating protein degradation in diseases in which proteasome function is impaired [18,69,70,84]. A fundamental question raised by these findings is why the cell’s degradative capacity, especially its ability to destroy misfolded or damaged proteins, is not normally maintained at maximal levels to provide cells protection, and why the capacity to destroy such proteins is activated by cAMP and PKA during exercise and fasting. Presumably, maintaining proteasomes in a continually activated state has negative consequences and could lead to excessive degradation of some critical regulatory proteins.

The rapid enhancement of the cell’s capacity to degrade short-lived proteins during exercise is of particular physiological interest. Such proteins represent only a minor fraction of cell constituents; therefore, even their complete hydrolysis and metabolism of the constituent amino acids cannot provide a significant source of energy to a fasting or exercising organism. Perhaps, after exercise, the enhanced capacity to degrade misfolded proteins enables the muscles to eliminate proteins damaged mechanically by the repeated contractions or by free radicals generated by mitochondrial metabolism. Another attractive possibility would be that the PKA-accelerated degradation of short-lived regulatory proteins with exercise, early in fasting, or in various hormonal responses facilitates adaptive changes in the cell’s protein composition. As these cells adapt to new physiological conditions, it would seem advantageous to degrade certain preexistent regulatory proteins or critical enzymes that are deleterious under the new conditions. In such cells, cAMP and cGMP also stimulate via CREB the expression of new proteins which are more appropriate for these new conditions. More rapid elimination of some preexistent regulatory proteins could synergize with this enhancement of new gene expression to promote the cellular adaptation to the new physiological states.
